# Sex Difference in the Impact of Steatotic Liver Disease on Incident Hepatocellular Carcinoma in Patients with Chronic Hepatitis B

**DOI:** 10.3390/cancers18172880

**Published:** 2026-09-06

**Authors:** Wan-Jung Wu, Chih-Hsuan Tien, Chih-Lin Lin, Chun-Jen Liu, Jui-Ting Hu, Jyh-Ming Liou, Ming-Whei Yu

**Affiliations:** 1 Institute of Epidemiology and Preventive Medicine, College of Public Health, National Taiwan University, Taipei 10055, Taiwan; 2 Department of Gastroenterology, Ren-Ai Branch, Taipei City Hospital, Taipei 10629, Taiwan; 3 Division of Gastroenterology and Hepatology, Department of Internal Medicine, National Taiwan University Hospital, Taipei 10048, Taiwan; 4 Hepatitis Research Center, National Taiwan University Hospital, Taipei 100229, Taiwan; 5 Graduate Institute of Clinical Medicine, National Taiwan University College of Medicine, Taipei 10048, Taiwan; 6 Liver Center, Cathay General Hospital Medical Center, School of Medicine, Fu-Jen Catholic University College of Medicine, Taipei 24352, Taiwan; 7 Department of Internal Medicine, National Taiwan University College of Medicine, Taipei 10051, Taiwan; 8 Department of Internal Medicine, National Taiwan University Cancer Center, Taipei 106037, Taiwan

**Keywords:** chronic hepatitis B, diabetes, entecavir, gender, hepatocellular carcinoma, steatotic liver disease, tenofovir

## Abstract

**Background and Aims:** Until now, according to the EASL Clinical Practice Guidelines on the management of chronic hepatitis B (CHB), the relationship between steatotic liver disease (SLD) and the risk of hepatocellular carcinoma (HCC) in CHB patients still yields conflicting results, with some data suggesting an increased risk of HCC, while several cohort studies have indicated a reduced risk in CHB patients with SLD. Sex is a predominant factor in the development and severity of chronic liver diseases. We aimed to understand the effect modification by sex in this association using a large population-based cohort study with long-term follow-up of patients with CHB. **Approach and Results:** This population-based study from Taiwan’s National Health Insurance Research Database (2009–2022) comprised two cohorts of patients with CHB aged 40–75 years: (1) 53,888 patients treated with entecavir or tenofovir, and (2) 525,109 antiviral-naive patients. HCC was identified in 4245 and 9491 patients in the treated and untreated cohorts, respectively. After adjusting for competing risk of death and relevant covariates, SLD was associated with a lower risk of HCC in males but a higher risk in females, and this sex difference (*p* < 0.0001 for sex–SLD interaction) was identified in both treated (subdistribution hazard ratio [SHR] [95% CI]: 0.85 [0.74–0.96] in males and 1.42 [1.17–1.73] in females) and untreated (0.75 [0.67–0.84] in males and 1.34 [1.14–1.56] in females) patients. Using propensity score matching with stratification by sex, further accounting for differences in medication use between SLD and non-SLD groups, yielded consistent results. The excess risk of HCC with SLD among females was mainly in those aged≥55 years, and even existed in the presence of diabetes, whereas among males, SLD exhibited an inverse association with HCC, regardless of age or diabetes. **Conclusions:** There was a sex disparity in the association between SLD and CHB-related HCC. Future studies and management of SLD for CHB should consider the sex disparity.

## Simple Summary

The association between steatotic liver disease (SLD) and hepatocellular carcinoma (HCC) remains controversial and complex in patients with chronic hepatitis B (CHB), and whether the impact of SLD differs by sex is unclear. This large population-based study of CHB patients revealed that a recorded diagnosis of SLD was associated with a significant 15–30% lower risk of HCC in males but a significant 30–40% higher risk of HCC in females. This gender difference was found in both antiviral-treated and untreated patients, even among patients with diabetes; but in females, the excess risk of HCC from SLD was more prominent in older age (≥55 years). These results suggest a need to closely evaluate for SLD and advanced liver diseases in routine care of female CHB patients over age 55.

## 1. Introduction

Steatotic liver disease (SLD), encompassing metabolic dysfunction-associated steatotic liver disease (MASLD), formerly referred to as nonalcoholic fatty liver disease (NAFLD), is increasingly becoming a common cause of chronic liver disease worldwide, while chronic hepatitis B (CHB) remains the leading cause of hepatocellular carcinoma (HCC), accounting for 39% of HCC cases [1,2]. In Asia-Pacific regions where CHB is endemic, the impact of SLD on the natural course of CHB is a growing concern. A number of studies have investigated the relationship between SLD and HCC in patients with CHB, but the long-term risk of HCC in patients with CHB and SLD is complicated and not fully understood [3,4,5,6,7,8,9,10]. Among patients with CHB, those with SLD have a higher risk of metabolic disease complications, and diabetes and a higher burden of metabolic risk factors have been linked to increased risk of HCC [5,11,12,13]. However, CHB patients with concurrent SLD tend to have lower viral activity, which potentially leads to lower subsequent risk of HCC [4,5,6,8,10,13,14]. Additionally, several studies have shown a reduced risk of HCC among CHB patients with SLD detected by ultrasound-based methods compared to those without SLD [3,4,5,6,7,9]. In contrast, other studies based on liver biopsy cohorts of CHB patients, which were expected to have higher disease severity, demonstrated the opposite impact of SLD on HCC risk [10].

Sex is a predominant factor for the development and severity of chronic liver diseases [15]. Specifically, there is a substantial sex disparity in CHB-related HCC incidence, often with a male-to-female ratio of 4:1 or higher [16]. Epidemiological research has shown that androgen concentrations and androgen receptor activity are associated with CHB-related HCC incidence [17,18]. In experimental studies, androgen and estrogen signaling exert opposite regulatory effects on hepatitis B virus (HBV) RNA transcription by competing and interacting at the viral enhancer I [19,20,21]. The HBV X protein could cooperate with the androgen receptor signaling pathway to enhance hepatocarcinogenesis [19,21]. Moreover, there are differences between the sexes in lipid metabolism and cardiometabolic risk [22,23]. Indeed, substantial experimental data indicate that sex hormones influence mechanisms involved in the development and progression of MASLD [23]. The concentrations of sex hormones change with age, and mounting epidemiological evidence suggests that the decline in estrogen levels during menopause is associated with increased risk of HCC [24].

However, because of the male predominance in HCC incidence in CHB patients, females were often underrepresented, or HCC was mostly detected in males in previous studies regarding the impact of SLD. This makes it difficult to perform separate analyses of males and females because of insufficient sample size, leading to a lack of understanding of how SLD affects females differently in CHB patients. To our knowledge, this is the first and largest study to examine population-based incidence of HCC in association with SLD by sex and in different age groups among CHB patients. Although current first-line nucleos(t)ide analogues (NUCs) suppress HBV replication effectively, the residual risk of HCC in treated patients is not negligible [25]. Thus, we analyzed data for patients with CHB treated with first-line NUCs and untreated patients.

## 2. Patients and Methods

### 2.1. Data Source

This retrospective, population-based, cohort study used data from Taiwan’s National Health Insurance Research Database (NHIRD), a continually updated repository of data maintained by the Taiwan government. NHIRD captures de-identified data from the cancer registry, the death registry, the registry for catastrophic illness patient database (RCIPD), outpatient visits, inpatient administration, and detailed prescription drug use and diagnoses. The database was set up in 1997 and encompasses inpatient and outpatient data on 99% of Taiwan’s population of 24 million people. International Classification of Diseases (ICD) codes are used to define diseases in the database. The NHIRD has been used for many epidemiological studies of hepatic outcomes, and its validity has been reported [25,26,27,28]. Definitions of diseases/ICD codes used in this study are provided in Table S1. This study was approved by the Research Ethics Committee of National Taiwan University and the ethical review board of Taiwan’s Ministry of Health and Welfare Data Science Center.

### 2.2. Study Population

**Figure 1:**
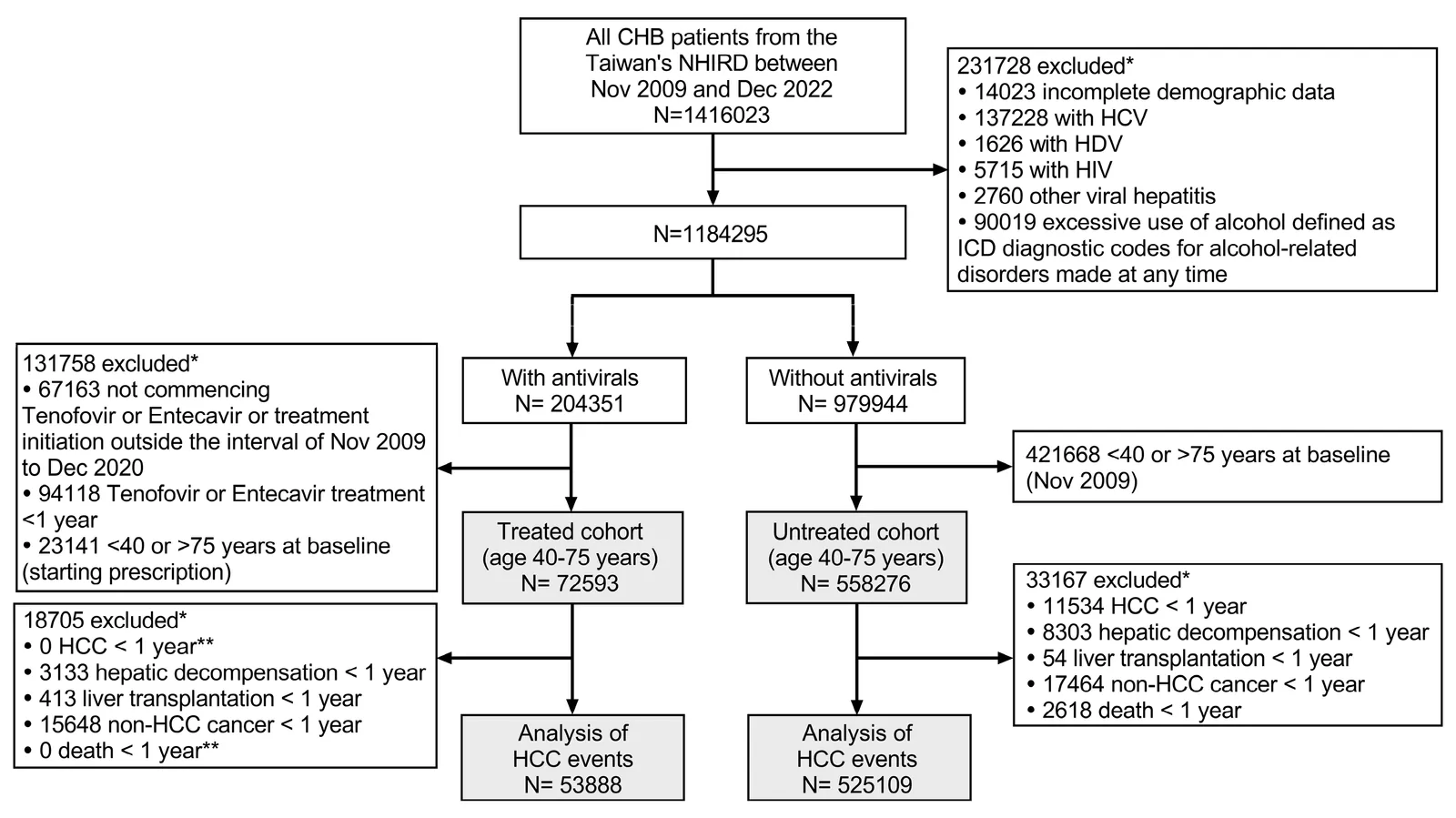
Flowchart of study patients selection. * A case can be excluded due to more than one criterion. Therefore, the total excluded cases in each step can be less than the sum of case numbers excluded by individual criterion. ** Eligible criteria of treated patients included treatment ≥ 1 year, meaning a 1-year waiting period for occurrence of HCC and death. NHIRD, National Health Insurance Research Database.

All patients diagnosed with ICD-9 or ICD-10 codes for HBV infection ≥ 2 times in outpatient or inpatient care were identified. We first excluded patients if they had incomplete demographic data; co-infected with HCV, HDV, or HIV; had other viral hepatitis; and/or had excessive use of alcohol, defined as ICD diagnostic codes for alcohol-related disorders made at any time. To perform two parallel analyses on patients with and without antiviral treatment, we constructed two distinct cohorts. The patient selection process is shown in Figure 1.

#### 2.2.1. Identification of Treated Cohort

Considering that the incidence of HCC and the prevalence of metabolic dysfunction-associated diseases significantly increase after the age of 40, we identified all patients with CHB aged 40–75 years who initiated tenofovir-based therapies (tenofovir disoproxil fumarate or tenofovir alafenamide) or entecavir for ≥1 year between November 2009 and December 2020. The period was chosen for the following two reasons: (1) since November 2009, the National Health Insurance reimbursement criteria for anti-HBV treatment were expanded to include high serum HBV DNA and persistently elevated ALT levels, and (2) to ensure a follow-up of ≥2 years for HCC events until December 2022. The general management, treatment indications, and discontinuation strategy during NUC therapy follow the guidelines of the Asian Pacific Association for the Study of the Liver. In principle, reimbursement is finite, with a maximum of 3 years. The reimbursement criteria for retreatment are similar to those for treatment initiation [25].

#### 2.2.2. Identification of Untreated Cohort

Because the criteria for the reimbursement of anti-HBV therapy changed in Taiwan in November 2009, the untreated cohort was selected among CHB patients who were still alive in November 2009, never treated with NUCs or interferon prescription during the study period because they did not meet the criteria for anti-HBV therapy, and aged 40–75 years at baseline.

To assess the incidence of HCC, patients were further excluded from the analysis if they had cancer (including HCC), hepatic decompensation, or liver transplantation before or within 1 year after baseline, or had less than 1 year of follow-up.

Incidence analyses of nonliver events further excluded patients developing the specific outcome before or within 1 year after baseline (Figure S1).

### 2.3. Variables and Endpoints

We identified patients with a diagnosis of SLD before or at baseline using ICD-9 code 571.8 and ICD-10 codes K75.8, K76.0, which have been widely adapted in previous studies based on large electronic health record databases, and have been validated with a high positive predictive value of 95.8% in the NHIRD setting (Table S1) [28,29]. The primary endpoint was incident HCC, defined as a hospital admission with diagnostic codes for HCC and enrollment in the National Cancer Registry or RCIPD. Secondary endpoints were relevant nonliver events, including diabetes, hypertension, and cardiovascular disease (coronary heart disease [CHD] and stroke). Compensated cirrhosis was identified by the presence of ICD codes for cirrhosis with no decompensating events (variceal bleeding, ascites, hepatic encephalopathy, hepatorenal syndrome or spontaneous bacterial peritonitis), as defined previously [25].

### 2.4. Statistical Analysis

Descriptive statistics were used to summarize the patients’ characteristics at baseline by SLD in the treated and untreated cohorts. Start of follow-up was considered to be the onset of NUCs treatment for the treated and November 2009 for the untreated patients. We calculated the follow-up time from the start date to the date the outcome occurred, the date of death, or December 2022, whichever came first. We estimated cumulative incidence functions of HCC with adjustment for the competing risk of death, and Gray’s test was used to compare cumulative incidence functions between groups. Subdistribution hazard ratios (SHRs) with 95% confidence intervals (CIs) were obtained from the Fine–Gray model with adjustment for competing deaths. Given the focus of effect modification by sex on the risk of HCC in relation to SLD, we conducted separate analyses for males and females and compared the cumulative incidence functions and SHRs of SLD for males vs. females. Variables included in the multivariable models had potential association with the outcomes of interest from prior reports. The effect modification by age, sex, and comorbidities on the association between SLD and outcomes was assessed by including product terms for interaction with SLD in multivariable models. Statistical significance was defined as having a 2-tailed *p* < 0.05. All analyses were conducted with SAS 9.4 software (SAS Institute, Cary, NC, USA).

### 2.5. Sensitivity Analysis

To test the robustness of the sex-specific effect of SLD for HCC in the original cohort, we used propensity score matching (PSM) analysis to match baseline characteristics between SLD and non-SLD groups to minimize the potential for confounding bias. The PSM analyses were performed by treatment and sex. A propensity score for estimating the probability of SLD was developed using a logistic regression model including age, comorbidities, and medications (details shown in Table S2). In constructing the PSM cohort, a nearest neighbor 1:2 (SLD vs. non-SLD) matching scheme was applied with a caliper width of 0.2 and an absolute standardized difference (ASD) of less than 0.1.

## 3. Results

### 3.1. Baseline Characteristics

We categorized the patients with CHB into treated (*n* = 53,888; mean [SD] age, 53.6 [8.7] years; 16,659 [30.9%] female; and 14,083 [26.1%] cirrhosis) and untreated (*n*= 525,109; mean [SD] age, 51.5 [8.5] years; 252,575 [48.1%] female; and 18,102 [3.5%] cirrhosis) cohorts. The patients’ baseline characteristics are shown in Table 1 and Table S3 (for the eight strata of the study population by treatment, sex, and SLD in the original and the PSM cohorts). In both cohorts, patients with SLD were more likely to have metabolic comorbidities than those without SLD (all *p* < 0.0001).

### 3.2. Primary Endpoint

During follow-up (mean [SD] years, 6.9 [3.3] in treated and 12.7 [1.7] in untreated cohorts), 4245 (7.9%) subjects developed HCC, and there were 3901 (7.2%) deaths in the treated cohort, whereas 9491 (1.8%) subjects developed HCC and there were 37,609 (7.2%) deaths in the untreated cohort. The treated cohort had a higher incidence (per 1000 person-years) of HCC compared with untreated patients (11.35 [95% CI, 11.02–11.70] vs. 1.43 [95% CI, 1.40–1.45], *p* < 0.0001). Comparing males with females, the incidence of HCC was significantly higher in males among both treated (12.57 [12.14–13.01] vs. 8.60 [8.08–9.15], *p* < 0.0001) and untreated (2.17 [2.12–2.22] vs. 0.64 [0.61–0.66], *p* < 0.0001) patients. Male sex was positively and independently associated with incident HCC in the two cohorts; however, this association was stronger among younger than older (≥55 years) patients (adjusted SHRs [95% CI]: 2.07 [1.78–2.40] vs. 1.50 [1.38–1.62] in treated cohort; 5.05 [4.61–5.54] vs. 2.79 [2.63–2.96] in untreated cohort) (Table S4).

Table 2 presents the risk of HCC according to sex and SLD in treated and untreated patients, respectively. For the treated cohort, in males, the incidence (per 1000 person-years) of HCC was significantly lower among patients with SLD compared with those without SLD (9.67 [8.54–10.94] vs. 12.89 [12.44–13.36], *p* < 0.0001), but in females, the incidence rate was significantly higher among patients with SLD than those without SLD (11.84 [9.87–14.21] vs. 8.29 [7.76–8.87], *p* = 0.0018). After adjusting for age, metabolic comorbidities, cirrhosis, and NUCs treatment (tenovofir vs. entecavir), SLD was associated with a 15% (SHR 0.85 [0.74–0.96]; *p* = 0.0114) lower risk of HCC in males, but in females, a 42% (SHR 1.42 [1.17–1.73]; *p* = 0.0003) higher risk of HCC for SLD vs. no SLD was found. There was significant effect modification by sex on the relationship between SLD and HCC (*p* < 0.0001 for interaction).

**Figure 2:**
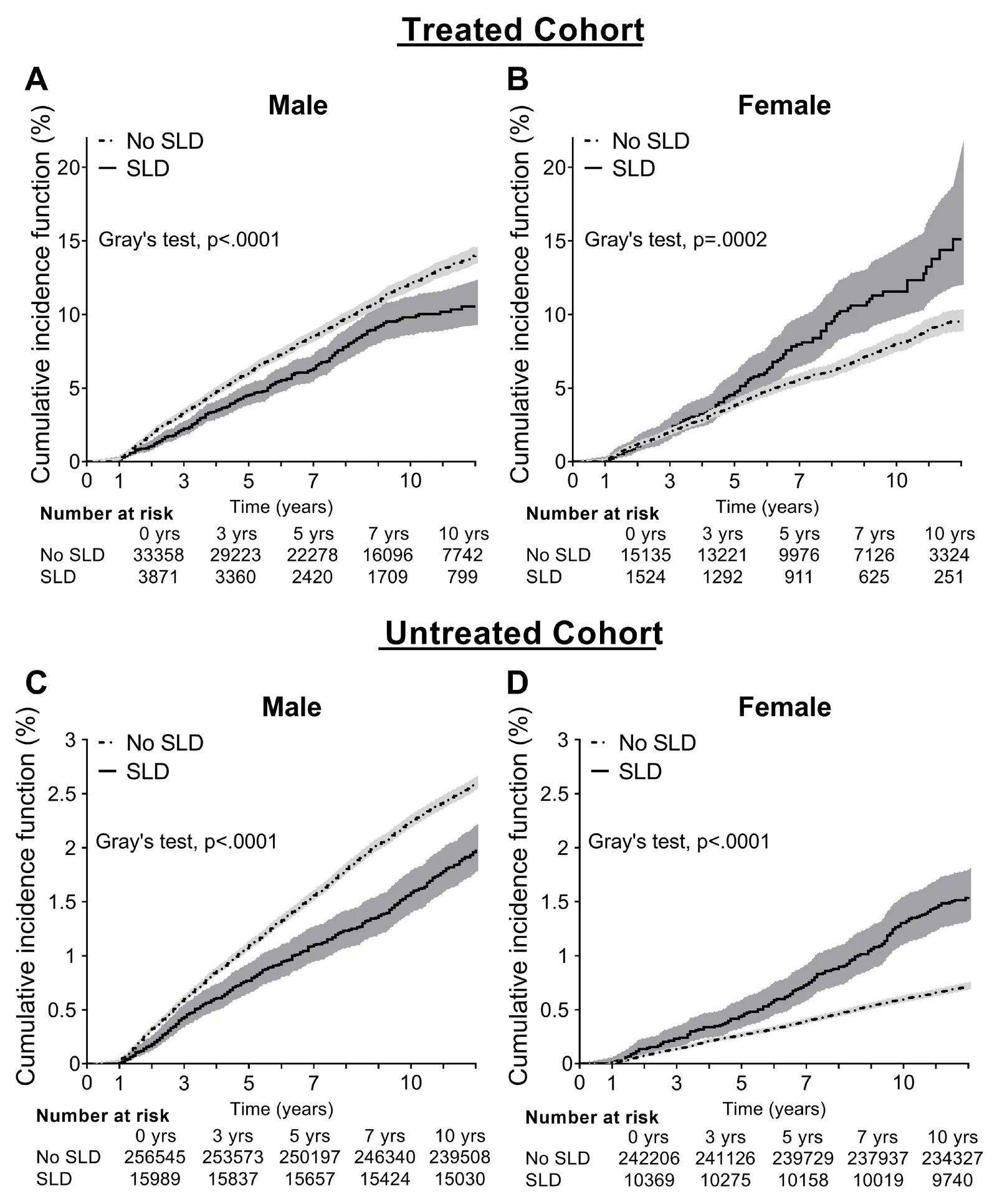
Cumulative incidence functions (95% CIs) of HCC between CHB patients with and without SLD after adjustment for competing risk of death. (**A**) Treated cohort: male. (**B**) Treated cohort: female. (**C**) Untreated cohort: male. (**D**) Untreated cohort: female.

We further examined the incidence rate of HCC among untreated patients according to sex and SLD. Consistent with findings from the analysis of the treated cohort, after adjusting for potential confounders, we also found SLD to be associated with a 25% (SHR 0.75 [0.67–0.84]; *p* < 0.0001) lower risk of HCC in males; but in females, there was a 34% (SHR 1.34 [1.14–1.56]; *p* = 0.0003) higher risk of HCC when comparing SLD vs. no SLD (Table 2). The SLD risk for HCC significantly differed by sex (*p* < 0.0001 for interaction). The cumulative risks of HCC for SLD vs. non-SLD according to sex are shown in Figure 2.

After PSM, the baseline clinical characteristics were adequately balanced between SLD and non-SLD groups, as demonstrated by an ASD of <0.1 in both male and female patients (Table S3). The results after PSM were similar to those observed from the original cohorts, and the sex difference in the association between SLD and HCC persisted (SHR [95% CI], treated cohort: 0.84 [0.72–0.97] and 1.35 [1.07–1.71] for males and females, respectively; untreated cohort: 0.74 [0.65–0.84] and 1.33 [1.10–1.62] for males and females, respectively) (Table 2).

### 3.3. Sex-Specific Risk of HCC Associated with SLD by Subgroups

**Figure 3:**
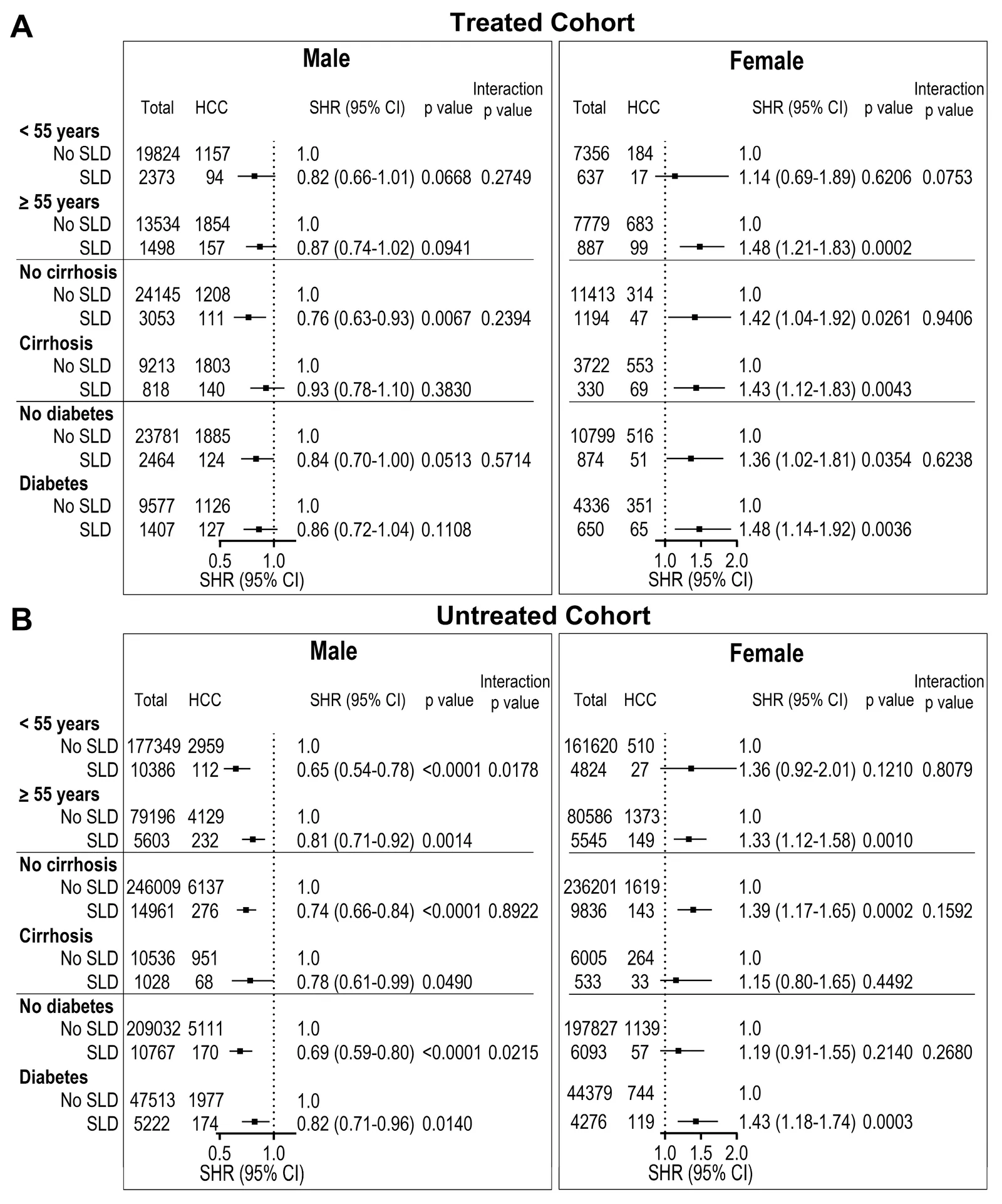
Subdistribution hazard ratios (95% CIs) of HCC for SLD vs. no SLD in CHB patients by sex, stratified by age, compensated cirrhosis, and diabetes at baseline. Subdistribution hazard ratios (SHRs) were adjusted for age, first-line NUCs (tenofovir vs. entecavir) (if treated cohort), and baseline comorbidities (compensated cirrhosis, diabetes, hyperlipidemia, and hypertension). (**A**) Treated cohort. (**B**) Untreated cohort.

We further investigated whether the gender effect in the association between SLD and HCC among CHB patients may be related to age, diabetes, and compensated cirrhosis at baseline (Figure 3). In both treated and untreated patients, there are distinct patterns for the association between sexes. In males, SLD was associated with lower risk of HCC in all subgroups, with a stronger association observed in the untreated (SHR: 0.65–0.82) than in the treated cohort (SHR: 0.76–0.93). In females, SLD was associated with a higher risk of HCC, and in the treated cohort, statistical significance was reached in nearly all subgroups (SHR: 1.36–1.48), except for younger female patients (<55 years). Although the positive association between SLD and HCC was also observed across all subgroups in untreated female patients, this association just reached statistical significance for older (SHR = 1.33), no cirrhosis (SHR = 1.39), and diabetes (SHR = 1.43) subgroups.

### 3.4. Secondary Outcomes

We evaluated five nonliver outcomes: diabetes, hypertension, cardiovascular disease, CHD, and stroke (Table S5). In the untreated cohort with a very large sample size, after adjusting for age and metabolic comorbidities, SLD was associated with a higher risk of diabetes, hypertension, cardiovascular disease, and CHD, with a significant positive association observed for SLD with the four metabolic diseases in both sexes (all *p* < 0.005). Similar associations between SLD and these four metabolic diseases were found in the treated cohort; however, the risk of SLD just reached statistical significance for diabetes and cardiovascular disease/CHD in male patients. There was no evidence for a difference in the direction of association between SLD and nonliver outcomes by sex.

## 4. Discussion

### 4.1. Main Findings

Although SLD is increasingly common among patients with CHB, it is uncertain whether SLD in such patients affects the incidence of HCC and whether the association is modified by sex [3,4,5,6,7,8,9,10,13]. In a population-scale nationally representative cohort of Taiwan involving 53,888 current first-line NUCs-treated and 525,109 untreated patients with CHB, with up to 13 years of follow-up, findings suggest that a recorded diagnosis of SLD was associated with a 15–30% reduction in HCC risk in males; in contrast, SLD in females with CHB was associated with a 30–40% increase in HCC risk, after multivariable adjustment or PSM, and a significant interaction between sex and SLD to HCC was identified in both the treated and untreated cohorts. The increased risk of HCC in female patients with SLD was mainly found in those over age 55 years. In addition, the risk of HCC in female patients with SLD appears to be increased even in those with baseline diabetes, which is a risk factor for HCC and is strongly associated with greater SLD prevalence and severity [5,12,30,31], while in male patients, there was an inverse association between SLD and HCC even in those with diabetes.

### 4.2. Comparison with Other Studies

Most studies that evaluated hepatic steatosis/NAFLD using ultrasound-based techniques have shown a lower risk of HCC among CHB patients with hepatic steatosis than those without hepatic steatosis, but there is some degree of inconsistency between studies, and none of these studies reported a sex-specific effect of SLD on HCC [3,4,5,6,7,9]. In the earliest study from Taiwan, including 2903 treatment-naïve male CHB patients and a median follow-up of 14.7 years, hepatic steatosis detected by ultrasonography was associated with an adjusted HR (aHR) of 0.24 [3]. Two subsequent studies based on parts of the same database yielded a similarly inverse association after accounting for HBsAg seroclearance and SLD-related genetic variants [4,5]. Moreover, in a study involving 6786 Asian CHB patients (63.5% male, 37.6% treated) from the United States and Taiwan and a mean follow-up of 10.9 years, hepatic steatosis detected by ultrasonography was associated with a reduced risk of HCC in antivirals-treated patients (aHR = 0.21) but not in untreated patients [6]. The inverse association of hepatic steatosis with HCC was also reported from two recent studies conducted in CHB patients recruited with transient elastography assessment [7,9]. In a study of 1823 Korean CHB patients under antiviral treatment, after 6.4 years of follow-up, a lower HCC risk was found to be associated with controlled attenuation parameter (CAP)-defined hepatic steatosis (≥222 dB/m) (aHR = 0.47), but this association was limited to 382 (70.9% male) patients with advanced fibrosis [7]. In another study of 2403 CHB patients (55.6% male, 57.1% treated) from Hong Kong with a median follow-up of 3.9 years, CAP-diagnosed steatosis (≥248 dB/m) was associated with reduced risk of HCC, showing an aHR of 0.41 after PSM for liver stiffness and other significant clinical variables. However, this study was based on only 48 incident HCC cases (75% male, 91.7% treated) [9]. Collectively, our findings of an inverse association between SLD and HCC in male CHB patients are generally in line with previous studies where males significantly outnumbered females [3,4,5,6,7,8,9].

Hepatic steatosis or MASLD has also been associated with lower HBV activity, including lower serum HBV DNA levels and higher rates of HBsAg seroclearance [4,5,6,8,10,13,14]. Entecavir and tenofovir are highly effective antivirals for suppressing HBV [25], which might lessen any potential association between SLD and HCC due to an inverse association between SLD and viral replication. The risk of HCC persists long after NUC treatment, and the impact of SLD on the treatment outcome in CHB patients has not been well established. In our study, similar patterns of sex-specific effects of SLD with HCC were found for patients under long-term NUC treatment, particularly entecavir- or tenofovir-based therapies, and for untreated patients, implying the generalizability of our findings.

Additionally, SLD has been associated with various metabolic disorders and increased risk of developing diabetes in people with and without CHB [5,13,14,30,32]. Diabetes is an important risk factor for HCC [5,12,30,31]. Previous studies have indicated a complex bidirectional association between SLD and diabetes [33]. However, while HCC risk in the presence of SLD among patients with CHB has been examined by many studies [3,4,5,6,7,8,9,10], evidence is lacking as to whether the presence of SLD confers an additional risk of HCC in patients with diabetes. In this study, we aimed to resolve some of the current uncertainties about the influence of SLD on HCC in higher risk groups, such as patients with diabetes or cirrhosis, and found that SLD still showed a differential effect in males and females among patients with baseline diabetes, where SLD was associated with a reduced risk in males but a statistically significant increased risk in females.

### 4.3. Potential Mechanisms for Sex Difference

Our results showed that SLD was significantly associated with higher rates of diabetes, hypertension, and cardiovascular disease in CHB patients, with similar associations observed for males and females. However, it is evident that gender disparity exists in the association between SLD and HCC. CHB and SLD are distinct causes of chronic liver disease, leading to hepatocarcinogenesis through different mechanisms: SLD involves dysregulated fatty acid metabolism and lipotoxicity [34], while CHB relies on viral DNA integrations, HBx protein-driven multiple oncogenic pathways, and immune response-mediated chronic inflammation [35]. It is well documented that HBV-related HCC is predominant in males [16]. In experimental studies, androgens demonstrated a synergistic oncogenic effect with HBV in males, which is not seen in females [19,20,21]. Among male HBV carriers, both higher serum levels of testosterone and more active androgen receptor gene alleles have been associated with an increased risk of HCC [17,18]. Furthermore, the presence of SLD or MASLD in CHB patients has been associated with a higher likelihood of HBsAg seroclearance [4,5,6,8,10,13,14]. The beneficial effect of SLD on HBsAg seroclearance might be stronger in males than females, although previous studies have reported somewhat heterogeneous results regarding the effect of sex on HBsAg seroclearance [14,36]. Therefore, SLD might be more impactful for HCC development in female CHB patients. On the other hand, accumulating evidence has suggested that premenopausal women are generally protected from HCC risk, with an increase in the risk after menopause due to the change in estrogen levels [24]. A meta-analysis found that women had a lower risk of NAFLD than men, but among patients with NAFLD, women had a higher risk of progression to advanced fibrosis than men, especially after age 50 years [37]. In the present study, the SHRs for HCC associated with sex diminished with age. Our results also indicated a more noticeable and significant risk of HCC for female patients with SLD in older (≥55 years) than in younger age, suggesting the importance of menopausal status.

### 4.4. Strengths of This Study

The strengths of this large population-based study include long follow-up and the power to detect gender differences in the association between SLD and HCC among CHB patients, as imaging and liver biopsies often could not be conducted in such a large-scale epidemiological study. Furthermore, we conducted our study concurrently in two distinct cohorts of treated and untreated patients and observed a similar pattern of sex-specific effect of SLD on CHB progression to HCC, strengthening the important role of sex in the impact of SLD for the development of HBV-related HCC.

### 4.5. Study Limitations

Although we used specific ICD codes established by an expert panel to define SLD [29], which enabled the inclusion of large cohorts on a nationwide scale, using this approach would result in an underestimated prevalence of SLD, given that SLD often has no symptoms. However, the ICD codes for SLD have been validated with a high positive predictive value of 95.8% using the same database [28], which is in agreement with other validation studies of these codes in other countries [38]. In addition, as routine ultrasonography is used for HCC surveillance for CHB patients, even though a proportion of study patients in the no SLD group had undiagnosed SLD, this misclassification of SLD would typically bias the observed association between SLD and HCC towards the null. Moreover, SLD patients showed a significantly higher prevalence and incidence of metabolic disorders compared with those with no SLD. These findings also provided evidence justifying the credibility of the use of the diagnostic codes to define SLD in this study.

Another limitation is the lack of direct data on the severity of SLD. However, using the diagnosis of cirrhosis, which is closer to a practical point of view, we found a similarly significant 40% increase in HCC risk for female patients with or without baseline cirrhosis in the treated cohort with factors increasing the risk of HCC. Lastly, detailed laboratory and anthropometric data are not available; we cannot deeply investigate the mechanisms by which sex differences affect the impact of SLD on hepatocarcinogenesis.

## 5. Conclusions

We found evidence of significant sex differences in the association of SLD with CHB-related HCC risk. SLD was associated with a lower risk of HCC in males but a higher risk of HCC in females. The gender diversity in the impact of SLD on HCC was found in both treated and untreated patients with CHB, even among those with diabetes, but in females, the excess risk of HCC from SLD was more prominent in older age (≥55 years). Further studies with detailed data on metabolic risk factors and severity of SLD should be encouraged to verify our findings. If our findings are confirmed, screening for SLD and monitoring the progression of chronic liver disease are warranted in female CHB patients aged 55 years or older.

## Figures and Tables

**Table 1:** Baseline characteristics and follow-up outcomes by SLD in the treated and untreated cohorts.

	Treated Cohort (*n *= 53,888)			Untreated Cohort (*n *= 525,109)		
Characteristics	SLD (*n *= 5395)	No SLD (*n *= 48,493)	*p* Value	SLD (*n *= 26,358)	No SLD (*n *= 498,751)	*p* Value
**Baseline**						
Male sex, *n* (%)	3871 (71.8)	33,358 (68.8)	<0.0001	15,989 (60.7)	256,545 (51.4)	<0.0001
Female sex, *n* (%)	1524 (28.3)	15,135 (31.2)		10,369 (39.3)	242,206 (48.6)	
Age, years	53.8 (8.8)	53.6 (8.7)	0.3392	53.5 (8.7)	51.4 (8.5)	<0.0001
Comorbidities, *n* (%)						
Compensated cirrhosis	1148 (21.3)	12,935 (26.7)	<0.0001	1561 (5.9)	16,541 (3.3)	<0.0001
Diabetes	2057 (38.1)	13,913 (28.7)	<0.0001	9498 (36.0)	91,892 (18.4)	<0.0001
Hyperlipidemia	3334 (61.8)	22,466 (46.3)	<0.0001	16,505 (62.6)	169,170 (33.9)	<0.0001
Hypertension	2975 (55.1)	23,248 (47.9)	<0.0001	15,021 (57.0)	187,667 (37.6)	<0.0001
Cardiovascular disease	1727 (32.0)	12,356 (25.5)	<0.0001	9969 (37.8)	111,397 (22.3)	<0.0001
Coronary heart disease	1469 (27.2)	10,179 (21.0)	<0.0001	8692 (33.0)	93,622 (18.8)	<0.0001
Stroke	555 (10.3)	4156 (8.6)	<0.0001	3368 (12.8)	36,262 (7.3)	<0.0001
Chronic kidney disease	563 (10.4)	4585 (9.5)	0.0201	2467 (9.4)	28,298 (5.7)	<0.0001
COPD	967 (17.9)	7455 (15.4)	<0.0001	5728 (21.7)	69,835 (14.0)	<0.0001
Medication use, *n* (%)						
Metformin	1303 (24.2)	8414 (17.4)	<0.0001	4921 (18.7)	41,776 (8.4)	<0.0001
Statins	1723 (31.9)	11,293 (23.3)	<0.0001	7496 (28.4)	66,166 (13.3)	<0.0001
Aspirin	3858 (71.5)	33,455 (69.0)	0.0001	17,998 (68.3)	299,436 (60.0)	<0.0001
NSAIDs	4761 (88.3)	41,472 (85.5)	<0.0001	22,025 (83.6)	389,936 (78.2)	<0.0001
Hepatoprotective agent	4276 (79.3)	35,412 (73.0)	<0.0001	8600 (32.6)	76,293 (15.3)	<0.0001
Antiviral treatment, *n* (%)						
Tenofovir	1795 (33.3)	14,550 (30.0)	<0.0001			
Entecavir	3600 (66.7)	33,943 (70.0)				
Prior history of other NUCs	522 (9.7)	4416 (9.1)	0.1693			
Prior history of interferon	178 (3.3)	1147 (2.4)	<0.0001			
**Follow-up**						
Follow-up time, years ^a^	6.6 (3.2)	7.0 (3.3)	<0.0001	12.6 (1.8)	12.7 (1.7)	<0.0001
HCC, *n* (%)	367 (6.8)	3878 (8.0)	0.0020	520 (2.0)	8971 (1.8)	0.0386
Death, *n* (%)	332 (6.2)	3569 (7.4)	0.0012	2261 (8.6)	35,348 (7.1)	<0.0001

COPD, chronic obstructive pulmonary disease; HCC, hepatocellular carcinoma; NSAIDs, non-steroidal anti-inflammatory drugs; NUCs, nucleos(t)ide analogues; SLD, steatotic liver disease. Categorical variables were presented as number (percentage), whereas age and follow-up time were expressed as mean (SD). Statistical significance in differences between groups were examined by χ^2^ test for categorical variables and Student’s *t* test for continuous variables. ^a^ Calculated from baseline to the diagnosis of HCC, death, or end of follow-up (December, 2022), whichever came first.

**Table 2:** Incidence rates and SHRs for HCC according to SLD by sex in the treated and untreated cohorts.

	Original Cohort				PSM Cohort			
	Total	HCC	Incidence (95% CI) (1/1000 Person-Years)	SHR (95% CI)	Total	HCC	Incidence (95% CI) (1/1000 Person-Years)	SHR (95% CI)
**Treated** **cohort ^a,b^**								
Male								
No SLD	33,358	3011	12.89 (12.44–13.36)	1.0 (reference)	7742	602	11.26 (10.40–12.20)	1.0 (reference)
SLD	3871	251	9.67 (8.54–10.94)	0.85 (0.74–0.96)	3871	251	9.67 (8.54–10.94)	0.84 (0.72–0.97)
Female								
No SLD	15,135	867	8.29 (7.76–8.87)	1.0 (reference)	3048	183	8.91 (7.71–10.30)	1.0 (reference)
SLD	1524	116	11.84 (9.87–14.21)	1.42 (1.17–1.73)	1524	116	11.84 (9.87–14.21)	1.35 (1.07–1.71)
**Untreated cohort ^b,c^**								
Male								
No SLD	256,545	7088	2.20 (2.15–2.25)	1.0 (reference)	31,978	897	2.25 (2.11–2.40)	1.0 (reference)
SLD	15,989	344	1.71 (1.54–1.90)	0.75 (0.67–0.84)	15,989	344	1.71 (1.54–1.90)	0.74 (0.65–0.84)
Female								
No SLD	242,206	1883	0.61 (0.58–0.63)	1.0 (reference)	20,738	260	1.00 (0.88–1.12)	1.0 (reference)
SLD	10,369	176	1.35 (1.16–1.56)	1.34 (1.14–1.56)	10,369	176	1.35 (1.16–1.56)	1.33 (1.10–1.62)

HCC, hepatocellular carcinoma; PSM, propensity score matching; SHR, subdistribution hazard ratio; SLD, steatotic liver disease. ^a^ SHRs were adjusted for age, first-line NUCs (tenofovir vs. entecavir), and baseline comorbidities (compensated cirrhosis, diabetes, hyperlipidemia, and hypertension). ^b^ *p* < 0.0001 for interaction between sex and SLD in original cohort. ^c^ SHRs were adjusted for age and baseline comorbidities (compensated cirrhosis, diabetes, hyperlipidemia, and hypertension).

## Data Availability

The Taiwan National Health Insurance Administration and the Health and Welfare Data Science Center of the Taiwan Ministry of Health and Welfare provided the database used in this study. Since the data are subject to national data protection laws to ensure data privacy of the study participants, only investigators who receive approval of a proposal and sign a data access agreement can request access to the data.

## Supplementary Materials

The following supporting information can be downloaded at: https://www.mdpi.com/article/10.3390/cancers18172880/s1, Figure S1: Patient flowchart for analysis of secondary outcomes. Table S1: ICD codes and disease definitions used for this study. Table S2: Factors included in propensity score. Table S3: Baseline characteristics and follow-up outcomes according to SLD by sex and treatment status before and after PSM. Table S4: Multivariate analysis with the Fine–Gray model for the risk of HCC, overall and by age, in the treated and untreated cohorts. Table S5: Multivariable-adjusted subdistribution hazard ratios (SHRs) for nonliver events according to SLD, overall and by sex, in the treated and untreated cohorts.

## Abbreviations

The following abbreviations are used in this manuscript:

ASD	absolute standardized difference
CAP	controlled attenuation parameter
CHB	chronic hepatitis B
CHD	coronary heart disease
CI	confidence interval
HBV	hepatitis B virus
HCC	hepatocellular carcinoma
MASLD	metabolic dysfunction-associated steatotic liver disease
NAFLD	nonalcoholic fatty liver disease
NHIRD	National Health Insurance Research Database
NUCs	nucleos(t)ide analogues
PSM	propensity score matching
RCIPD	registry for catastrophic illness patient database
SHR	subdistribution hazard ratio
SLD	steatotic liver disease
