# Supplementary material for: Sex Difference in the Impact of Steatotic Liver Disease on Incident Hepatocellular Carcinoma in Patients with Chronic Hepatitis B
Source: Cancers (Basel). 2026 Sep 6;18(17):2880. doi: 10.3390/cancers18172880 (PMC13565318; doi:10.3390/cancers18172880)
Supplement: Supplementary file 1 [file cancers-18-02880-s001.zip › cancers-4496255-supplementary.pdf]

Sex difference in the impact of steatotic liver disease on incident hepatocellular carcinoma in patients with chronic hepatitis B

Wan-Jung Wu, Chih-Hsuan Tien, Chih-Lin Lin, Chun-Jen Liu, Jui-Ting Hu, Jyh-Ming Liou, Ming-Whei Yu

Table of contents

Table S1……………………………………………………………….2
Table S2……………………………………………………………….3
Table S3……………………………………………………………….4-5
Table S4……………………………………………………………….6
Table S5……………………………………………………………….7-8
Fig. S1………………………………………………………………....9

Table S1. ICD codes and disease definitions used for this study.

	ICD-9 code	ICD-10 code	Additional conditions
Cancer	14x-20x	Cxx	
Hepatocellular carcinoma	155 (excluding 155.1)	C22 (excluding C22.1)	
Chronic hepatitis B	070.2, 070.3, V02.61	B16.0, B16.1, B16.2, B16.9, B18.0, B18.1, B19.10, B19.11, Z22.51	≥2 diagnostic codes from outpatient and/or inpatient care records.
Hepatitis C	070.41, 070.44, 070.51, 070.54, 070.7, V02.62	B17.1, B18.2	
Hepatitis D	070.52	B17.0	
Other viral hepatitis	V02.69	Z22.59	
Human immunodeficiency virus	042	B20	
Cirrhosis	571.2, 571.5	K74.0, K74.1, K74.2, K74.60, K74.69	Identified by RCIPD or diagnostic codes from outpatient or inpatient care records.
Acute-on-chronic liver failure	570	K72.0	
Hepatic decompensation			Defined by ≥1 decompensating event (variceal bleeding, ascites, hepatic encephalopathy, hepatorenal syndrome or spontaneous bacterial peritonitis) combined with the ICD codes for cirrhosis or acute-on-chronic liver failure, or the presence of severe decompensated cirrhosis registered in the RCIPD.
Ascites	789.5	R18	Defined by diagnostic codes from outpatient or inpatient care records combined with diagnostic codes for cirrhosis or acute-on-chronic liver failure.
Variceal bleeding	456.0, 456.20	I85.01, I85.11	
Hepatic encephalopathy	572.2	K72.90, K72.91	
Hepatorenal syndrome	572.4	K76.7	
Spontaneous bacterial peritonitis	567	K65.2	
Alcohol-related disorders	291.0-291.5, 291.81, 291.89, 291.9, 303.00-303.03, 303.90-303.93, 305.0, 357.5, 425.5, 535.3, 571.0-571.3, 790.3, 980.0, V11.3, V65.42, V79.1	E52, F10, G62.1, I42.6, K29.2, K70.0-K70.4, K70.9, T51, Z50.2, Z71.4, Z72.1	Defined by one inpatient or ≥2 outpatient diagnostic codes.
Diabetes	250	E10-E14	Defined by one inpatient or ≥2 outpatient diagnostic codes or any use of medication.
Hypertension	401-405	I10-I15	
Hyperlipidemia	272.0-272.4	E78.0-78.5	
Steatotic liver disease	571.8	K75.8, K76.0	Defined by one inpatient or ≥2 outpatient diagnostic codes.
Cardiovascular disease (coronary heart disease and stroke)	410-414, 428, 429, 430-438, 440, 441	I20-I25, I50, I60-I69, I70, I71, G45, G46	
Coronary heart disease	410-414, 428, 429, 440, 441	I20-I25, I50, I70, I71	
Stroke	430-438	I60-I69, G45, G46	
Chronic obstructive pulmonary disease	491, 492, 493.2, 496	J41-J44	
Chronic kidney disease	403.00, 403.01, 403.10, 403.11, 403.90, 403.91, 404.00-404.03, 404.10-404.13, 404.90-404.93, 582, 583, 585, 586, 588.0, V42.0, V45.11, V45.12, V56.0, V56.1, V56.2, V56.31, V56.32, V56.8	I12.0, I12.9, I13.0, I13.10, I13.11, I13.2, N03, N05, N18.1-N18.9, N19, N25.0, Z49, Z94.0, Z99.2	

RCIPD, registry for catastrophic illness patient database.

Table S2. Factors included in propensity score

Age (continuous variable)
Comorbidities
Compensated cirrhosis
Diabetes
Hyperlipidemia
Hypertension
Cardiovascular disease
Chronic kidney disease
Chronic obstructive pulmonary disease
Medications
Metformin
Statins
Aspirin
Non-steroidal anti-inflammatory drugs
Hepatoprotective agent
Antiviral treatment (for the treated cohort only)
Tenofovir or Entecavir
Prior history of other NUCs
Prior history of interferon

Table S3. Treated cohort: Baseline characteristics and follow-up outcomes according to SLD by sex before and after PSM

	Male						Female					
	Original cohort			PSM cohort			Original cohort			PSM		
Characteristics	SLD	No SLD	p value	SLD	No SLD	ASD[a]	SLD	No SLD	p value	SLD	No SLD	ASD[a]
**Treated patients, n**	3871	33358		3871	7742		1524	15135		1524	3048	
**Baseline**												
Age, mean (SD), years	52.6 (8.5)	52.9 (8.6)	0.0166	52.6 (8.5)	52.3 (8.4)	0.03312	56.7 (8.7)	55.2 (8.9)	<0.0001	56.7 (8.7)	57.0 (8.5)	0.02776
Comorbidities, n (%)												
Compensated cirrhosis	818 (21.1)	9213 (27.6)	<0.0001	818 (21.1)	1638 (21.2)	0.00060	330 (21.7)	3722 (24.6)	0.0108	330 (21.7)	637 (20.9)	0.01791
Diabetes	1407 (36.4)	9577 (28.7)	<0.0001	1407 (36.4)	2766 (35.7)	0.01328	650 (42.7)	4336 (28.7)	<0.0001	650 (42.7)	1265 (41.5)	0.02423
Hyperlipidemia	2330 (60.2)	15196 (45.6)	<0.0001	2330 (60.2)	4645 (60.0)	0.00392	1004 (65.9)	7270 (48.0)	<0.0001	1004 (65.9)	2003 (65.7)	0.00337
Hypertension	2074 (53.6)	15987 (47.9)	<0.0001	2074 (53.6)	4153 (53.6)	0.00129	901 (59.1)	7261 (48.0)	<0.0001	901 (59.1)	1799 (59.0)	0.00199
Cardiovascular disease	1171 (30.3)	8116 (24.3)	<0.0001	1171 (30.3)	2228 (28.8)	0.03313	556 (36.5)	4240 (28.0)	<0.0001	556 (36.5)	1055 (34.6)	0.04017
Coronary heart disease	991 (25.6)	6690 (20.1)	<0.0001	991 (25.6)	1870 (24.2)		478 (31.4)	3489 (23.1)	<0.0001	478 (31.4)	897 (29.4)	
Stroke	354 (9.1)	2654 (8.0)	0.0102	354 (9.1)	710 (9.2)		201 (13.2)	1502 (9.9)	<0.0001	201 (13.2)	387 (12.7)	
Chronic kidney disease	390 (10.1)	3118 (9.4)	0.1423	390 (10.1)	664 (8.6)	0.05060	173 (11.4)	1467 (9.7)	0.0383	173 (11.4)	326 (10.7)	0.02139
COPD	677 (17.5)	5158 (15.5)	0.0010	677 (17.5)	1243 (16.1)	0.03866	290 (19.0)	2297 (15.2)	<0.0001	290 (19.0)	518 (17.0)	0.05408
Medication use, n (%)												
Metformin	919 (23.7)	5999 (18.0)	<0.0001	919 (23.7)	1803 (23.3)	0.01115	384 (25.2)	2415 (16.0)	<0.0001	384 (25.2)	766 (25.1)	0.00163
Statins	1158 (29.9)	7594 (22.8)	<0.0001	1158 (29.9)	2293 (29.6)	0.00677	565 (37.1)	3699 (24.4)	<0.0001	565 (37.1)	1166 (38.3)	0.02583
Aspirin	2661 (68.7)	22297 (66.8)	0.0173	2661 (68.7)	5358 (69.2)	0.00995	1197 (78.5)	11158 (73.7)	<0.0001	1197 (78.5)	2441 (80.1)	0.03623
NSAIDs	3359 (86.8)	28019 (84.0)	<0.0001	3359 (86.8)	6780 (87.6)	0.02269	1402 (92.0)	13453(88.9)	0.0002	1402 (92.0)	2810 (92.2)	0.00670
Hepatoprotective agent	3084 (79.7)	24630 (73.8)	<0.0001	3084 (79.7)	6234 (80.5)	0.02023	1192 (78.2)	10782(71.2)	<0.0001	1192 (78.2)	2398 (78.7)	0.01060
Antiviral treatment, n (%)												
Tenofovir	1288 (33.3)	9955 (29.8)	<0.0001	1288 (33.3)	2541 (32.8)	0.00973	507 (33.3)	4595 (30.4)	0.0189	507 (33.3)	1018 (33.4)	0.00282
Entecavir	2583 (66.7)	23403 (70.2)		2583 (66.7)	5201 (67.2)		1017 (66.7)	10540(69.6)		1017 (66.7)	2030 (66.6)	
Prior history of other NUCs	390 (10.1)	3223 (9.7)	0.4112	390 (10.1)	718 (9.3)	0.02685	132 (8.7)	1193 (7.9)	0.2840	132 (8.7)	264 (8.7)	0.00000
Prior history of interferon	142 (3.7)	915 (2.7)	0.0010	142 (3.7)	271 (3.5)	0.00954	36 (2.4)	232 (1.5)	0.0142	36 (2.4)	82 (2.7)	0.02375
**Follow-up**												
Follow-up time[b]	6.7 (3.3)	7.0 (3.3)	<0.0001	6.7 (3.3)	6.9 (3.3)		6.4 (3.1)	6.9 (3.3)	<0.0001	6.4 (3.1)	6.7 (3.2)	
HCC, n (%)	251 (6.5)	3011 (9.0)	<0.0001	251 (6.5)	602 (7.8)		116 (7.6)	867 (5.7)	0.0029	116 (7.6)	183 (6.0)	
Death, n (%)	229 (5.9)	2616 (7.8)	<0.0001	229 (5.9)	570 (7.4)		103 (6.8)	953 (6.3)	0.4806	103 (6.8)	201 (6.6)	

Table S3 (*Continued*). Untreated cohort: Baseline characteristics and follow-up outcomes according to SLD by sex before and after PSM

	Male						Female					
	Original cohort			PSM cohort			Original cohort			PSM cohort		
Characteristics	SLD	No SLD	p value	SLD	No SLD	ASD[a]	SLD	No SLD	p value	SLD	No SLD	ASD[a]
**Untreated cohort, n**	15989	256545		15989	31978		10369	242206		10369	20738	
**Baseline**												
Age, mean (SD), years	52.0 (8.4)	51.2 (8.4)	<0.0001	52.0 (8.4)	52.0 (8.3)	0.00128	55.7 (8.8)	51.7 (8.5)	<0.0001	55.7 (8.8)	55.8 (8.8)	0.01443
Comorbidities, n (%)												
Compensated cirrhosis	1028 (6.4)	10536 (4.1)	<0.0001	1028 (6.4)	1765 (5.5)	0.04079	533 (5.1)	6005 (2.5)	<0.0001	533 (5.1)	819 (4.0)	0.06237
Diabetes	5222 (32.7)	47513 (18.5)	<0.0001	5222 (32.7)	10198 (31.9)	0.01786	4276 (41.2)	44379 (18.3)	<0.0001	4276 (41.2)	8513 (41.1)	0.00425
Hyperlipidemia	9788 (61.2)	86176 (33.6)	<0.0001	9788 (61.2)	19689 (61.6)	0.00736	6717 (64.8)	82994 (34.3)	<0.0001	6717 (64.8)	13566 (65.4)	0.01337
Hypertension	8822 (55.2)	101759 (39.7)	<0.0001	8822 (55.2)	17722 (55.4)	0.00494	6199 (59.8)	85908 (35.5)	<0.0001	6199 (59.8)	12495 (60.3)	0.00966
Cardiovascular disease	5403 (33.8)	56057 (21.9)	<0.0001	5403 (33.8)	10754 (33.6)	0.00366	4566 (44.0)	55340 (22.9)	<0.0001	4566 (44.0)	9092 (43.8)	0.00420
Coronary heart disease	4699 (29.4)	47012 (18.3)	<0.0001	4699 (29.4)	9157 (28.6)		3993 (38.5)	46610 (19.2)	<0.0001	3993 (38.5)	7878 (38.0)	
Stroke	1710 (10.7)	17957 (7.0)	<0.0001	1710 (10.7)	3530 (11.0)		1658 (16.0)	18305 (7.6)	<0.0001	1658 (16.0)	3146 (15.2)	
Chronic kidney disease	1326 (8.3)	14495 (5.7)	<0.0001	1326 (8.3)	2368 (7.4)	0.03492	1141 (11.0)	13803 (5.7)	<0.0001	1141 (11.0)	2122 (10.2)	0.02802
COPD	3363 (21.0)	38584 (15.0)	<0.0001	3363 (21.0)	6533 (20.4)	0.01574	2365 (22.8)	31251 (12.9)	<0.0001	2365 (22.8)	4655 (22.5)	0.00952
Medication use, n (%)												
Metformin	2715 (17.0)	23712 (9.2)	<0.0001	2715 (17.0)	5356 (16.8)	0.00690	2206 (21.3)	18064 (7.5)	<0.0001	2206 (21.3)	4395 (21.2)	0.00238
Statins	4214 (26.4)	33494 (13.1)	<0.0001	4214 (26.4)	8489 (26.6)	0.00486	3282 (31.7)	32672 (13.5)	<0.0001	3282 (31.7)	6621 (31.9)	0.00674
Aspirin	10361 (64.8)	148679 (58.0)	<0.0001	10361 (64.8)	20830 (65.1)	0.00695	7637 (73.7)	150757 (62.2)	<0.0001	7637 (73.7)	15369 (74.1)	0.00989
NSAIDs	13015 (81.4)	193153 (75.3)	<0.0001	13015 (81.4)	26284 (82.2)	0.01934	9010 (86.9)	196783 (81.3)	<0.0001	9010 (86.9)	18135 (87.5)	0.01520
Hepatoprotective agent	5766 (36.1)	49137 (19.2)	<0.0001	5766 (36.1)	11514 (36.0)	0.00128	2834 (27.3)	27156 (11.2)	<0.0001	2834 (27.3)	5601 (27.0)	0.00837
**Follow-up**												
Follow-up time[b]	12.6 (1.8)	12.6 (1.9)	0.0017	12.6 (1.8)	12.5 (2.1)		12.6 (1.8)	12.8 (1.3)	<0.0001	12.6 (1.8)	12.6 (1.8)	
HCC, n (%)	344 (2.2)	7088 (2.8)	<0.0001	344 (2.2)	897 (2.8)		176 (1.7)	1883 (0.8)	<0.0001	176 (1.7)	260 (1.3)	
Death, n (%)	1331 (8.3)	23007 (9.0)	0.0056	1331 (8.3)	3233 (10.1)		930 (9.0)	12341 (5.1)	<0.0001	930 (9.0)	1983 (9.6)	

ASD, absolute standardized difference; COPD, chronic obstructive pulmonary disease; HCC, hepatocellular carcinoma; NSAIDs, non-steroidal anti-inflammatory drugs; NUCs, nucleos(t)ide analogues; PSM; propensity score matching; SLD, steatotic liver disease.

Categorical variables were presented as number (percentage), whereas age and follow-up time were expressed in mean (SD). Statistical significance in differences between groups were examined by $\chi2$ test for categorical variables and Student's t test for continuous variables.

[a]An ASD <0.1 indicated good balance between SLD and no SLD groups. ASD was not calculated for variables not included in the propensity score.

[b]Calculated from baseline to the diagnosis of HCC, death, or end of follow-up (December 2022), whichever came first.

Table S4. Multivariate analysis with the Fine-Gray model for the risk of HCC, overall and by age, in the treated and untreated cohort

	Entire cohort			Age <55 years			Age ≥55 years		
Variable	SHR	95% CI	p value	SHR	95% CI	p value	SHR	95% CI	p value
**Treated cohort**									
Age, years	1.050	(1.046-1.054)	<0.0001	1.08	(1.06-1.09)	<0.0001	1.03	(1.02-1.04)	<0.0001
Male sex	1.63	(1.52-1.76)	<0.0001	2.07	(1.78-2.40)	<0.0001	1.50	(1.38-1.62)	<0.0001
Diabetes	1.27	(1.19-1.36)	<0.0001	1.31	(1.15-1.49)	<0.0001	1.25	(1.15-1.35)	<0.0001
SLD	0.97	(0.87-1.08)	0.6197	0.86	(0.71-1.05)	0.1340	1.04	(0.91-1.18)	0.5958
Hypertension	1.09	(1.02-1.17)	0.0140	1.12	(1.00-1.25)	0.0519	1.07	(0.98-1.16)	0.1259
Hyperlipidemia	0.83	(0.77-0.88)	<0.0001	0.83	(0.74-0.94)	0.0022	0.81	(0.75-0.87)	<0.0001
Compensated cirrhosis	3.65	(3.43-3.89)	<0.0001	4.46	(4.01-4.96)	<0.0001	3.20	(2.96-3.46)	<0.0001
Tenofovir vs. entecavir	0.76	(0.70-0.82)	<0.0001	0.78	(0.68-0.89)	0.0002	0.75	(0.69-0.83)	<0.0001
**Untreated cohort**									
Age, years	1.08	(1.07-1.08)	<0.0001	1.09	(1.08-1.10)	<0.0001	1.06	(1.059-1.07)	<0.0001
Male sex	3.38	(3.22-3.55)	<0.0001	5.05	(4.61-5.54)	<0.0001	2.79	(2.63-2.96)	<0.0001
Diabetes	1.45	(1.38-1.52)	<0.0001	1.55	(1.42-1.69)	<0.0001	1.41	(1.33-1.49)	<0.0001
SLD	0.89	(0.82-0.98)	0.0131	0.72	(0.61-0.86)	0.0002	0.97	(0.87-1.07)	0.5023
Hypertension	1.07	(1.03-1.13)	0.0024	1.09	(1.02-1.17)	0.0178	1.06	(1.00-1.13)	0.0439
Hyperlipidemia	0.65	(0.62-0.68)	<0.0001	0.57	(0.53-0.62)	<0.0001	0.67	(0.63-0.71)	<0.0001
Compensated cirrhosis	3.07	(2.88-3.26)	<0.0001	3.76	(3.38-4.17)	<0.0001	2.85	(2.65-3.07)	<0.0001

HCC, hepatocellular carcinoma; SHR, subdistribution hazard ratio; SLD, steatotic liver disease.

Table S5. Multivariable-adjusted subdistribution hazard ratios (SHRs) for nonliver events according to SLD, overall and by sex, in the treated and untreated cohort[a]

	Treated cohort[b]					Untreated cohort[c,d]				
	Total	Event	SHR	95% CI	p value	Total	Event	SHR	95% CI	p value
**Diabetes**										
Total										
No SLD	34002	4240	1.0	(reference)		399991	89354	1.0	(reference)	
SLD	3271	468	1.19	(1.08-1.30)	0.0005	16313	5008	1.21	(1.18-1.25)	<0.0001
Male										
No SLD	23367	2985	1.0	(reference)		205222	49606	1.0	(reference)	
SLD	2418	344	1.17	(1.05-1.31)	0.0054	10428	3186	1.17	(1.13-1.22)	<0.0001
Female										
No SLD	10635	1255	1.0	(reference)		194769	39748	1.0	(reference)	
SLD	853	124	1.20	(0.995-1.44)	0.0570	5885	1822	1.28	(1.22-1.34)	<0.0001
**Hypertension**										
Total										
No SLD	24376	5689	1.0	(reference)		299323	124011	1.0	(reference)	
SLD	2337	570	1.09	(0.996 -1.19)	0.0608	10705	5251	1.10	(1.07-1.13)	<0.0001
Male										
No SLD	16723	4156	1.0	(reference)		148085	68170	1.0	(reference)	
SLD	1735	438	1.08	(0.98-1.20)	0.1279	6770	3483	1.10	(1.06-1.13)	<0.0001
Female										
No SLD	7653	1533	1.0	(reference)		151238	55841	1.0	(reference)	
SLD	602	132	1.10	(0.91-1.32)	0.3277	3935	1768	1.08	(1.03-1.14)	0.0015
**Cardiovascular disease**										
Total										
No SLD	35567	3747	1.0	(reference)		380182	85739	1.0	(reference)	
SLD	3605	434	1.19	(1.08-1.32)	0.0006	15934	4576	1.09	(1.06-1.12)	<0.0001
Male										
No SLD	24832	2711	1.0	(reference)		196591	47936	1.0	(reference)	
SLD	2650	330	1.23	(1.10-1.38)	0.0004	10325	3010	1.09	(1.05-1.13)	<0.0001
Female										
No SLD	10735	1036	1.0	(reference)		183591	37803	1.0	(reference)	
SLD	955	104	1.07	(0.87-1.31)	0.5123	5609	1566	1.08	(1.02-1.14)	0.0042
**Coronary heart disease**										
Total										
No SLD	37882	2767	1.0	(reference)		399165	66270	1.0	(reference)	
SLD	3877	344	1.24	(1.10-1.38)	0.0002	17243	3812	1.11	(1.08-1.15)	<0.0001
Male										
No SLD	26352	2023	1.0	(reference)		206309	37760	1.0	(reference)	
SLD	2838	266	1.28	(1.13-1.45)	0.0001	11054	2515	1.11	(1.07-1.16)	<0.0001
Female										
No SLD	11530	744	1.0	(reference)		192856	28510	1.0	(reference)	
SLD	1039	78	1.08	(0.85-1.36)	0.5236	6189	1297	1.10	(1.04-1.17)	0.0008
**Stroke**										
Total										
No SLD	44028	2307	1.0	(reference)		459152	50828	1.0	(reference)	
SLD	4810	279	1.12	(0.99-1.27)	0.0730	22754	3239	1.04	(1.00-1.08)	0.0488
Male										
No SLD	30485	1567	1.0	(reference)		236830	26869	1.0	(reference)	
SLD	3498	189	1.10	(0.94-1.28)	0.2315	14150	1866	1.02	(0.97-1.07)	0.4777
Female										
No SLD	13543	740	1.0	(reference)		222322	23959	1.0	(reference)	
SLD	1312	90	1.19	(0.96-1.49)	0.1145	8604	1373	1.07	(1.01-1.13)	0.0190

SLD, steatotic liver disease.

[a]Incidence analysis of each outcome further excluded patients developing the specific outcome before or within 1 year after baseline. (please also see Fig. S1).
[b]Adjusted for age, sex (for total), first-line NUCs (tenofovir vs entecavir), and baseline comorbidities (diabetes, hyperlipidemia, and/or hypertension, as appropriate).
[C]Adjusted for age, sex (for total), and baseline comorbidities (diabetes, hyperlipidemia, and/or hypertension, as appropriate).
[d]$p<0.0001$ for interaction between sex and SLD in the risk of type 2 diabetes.

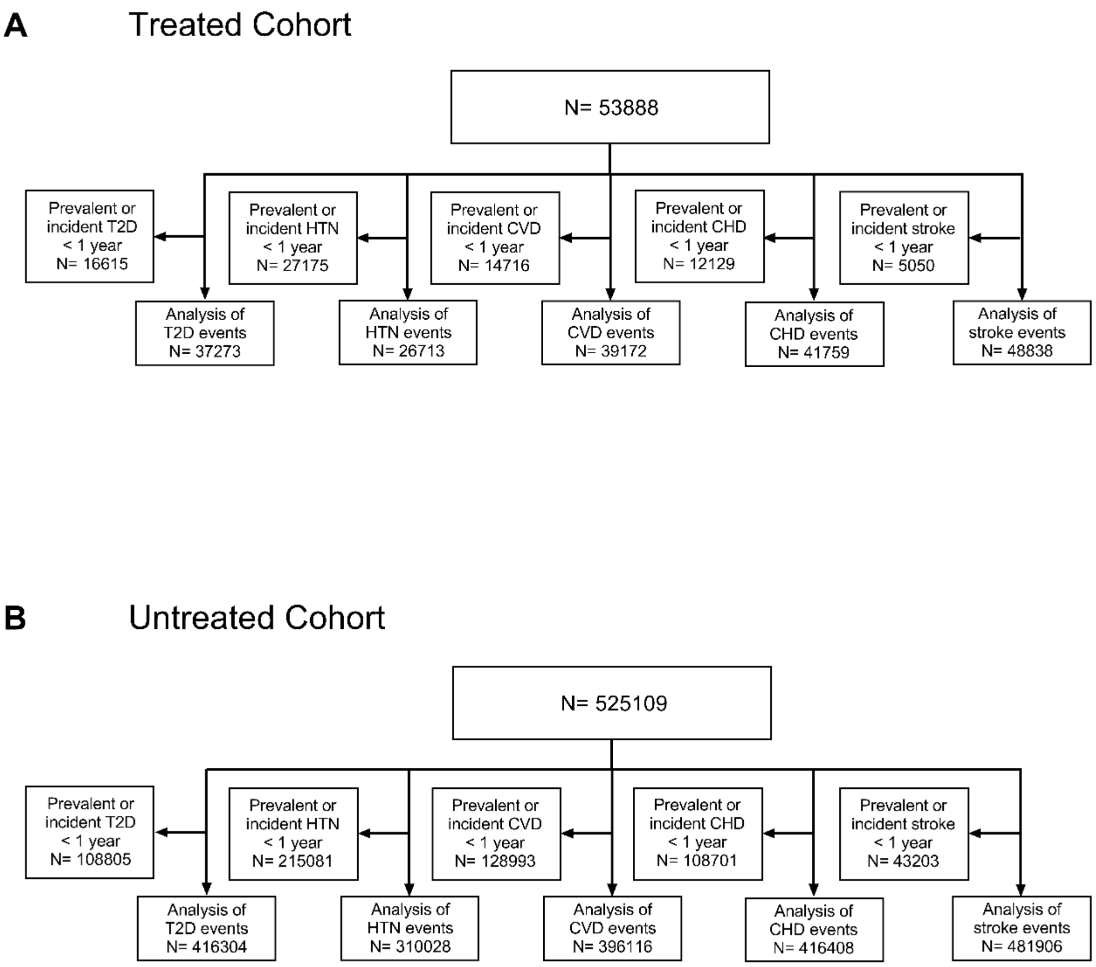

Fig. S1. Patients flowchart for analysis of secondary outcomes. (A) Treated cohort. (B) Untreated cohort. T2D, type 2 diabetes; HTN, hypertension; CVD, cardiovascular disease; CHD, coronary heart disease.
